# A Study on High-Precision Dimensional Measurement of Irregularly Shaped Carbonitrided 820CrMnTi Components

**DOI:** 10.3390/ma19081491

**Published:** 2026-04-08

**Authors:** Xiaojiao Gu, Dongyang Zheng, Jinghua Li, He Lu

**Affiliations:** 1 College of Mechanical Engineering, Shenyang Ligong University, Nanping Middle Road 6, Shenyang 110159, China; 2501620210@stu.sylu.edu.cn; 2 School of Mechanical Engineering, Dalian University of Technology, Dalian 116024, China; 13050505421@163.com; 3 Shenyang Institute of Engineering, No. 18 Puchang Road, Shenyang 110136, China; luhe@sie.edu.cn

**Keywords:** 820CrMnTi, dimensional measurement, adaptive exposure, Huber M-estimation, gradient boosting decision, machine vision

## Abstract

For irregularly shaped 820CrMnTi carburizing and nitriding parts, the challenges of high reflectivity-induced overexposure, low surface contrast, and interference from minute burrs in industrial online inspection are addressed in this paper. An innovative precision detection method integrating adaptive imaging and a dual-drive heterogeneous coupling model (RGFCN) is proposed. Such parts, due to surface photovoltaic characteristic changes caused by carburizing and nitriding heat treatment and the complex on-site lighting environment, are prone to local overexposure and “false out-of-tolerance” measurements caused by outlier sensitivity in traditional inspections. First, an innovative programmatic adaptive exposure control algorithm based on grayscale histogram feedback is introduced, which dynamically adjusts imaging parameters in real time to effectively suppress high-brightness overexposure under specific working conditions. Second, a novel adaptive main-axis scanning strategy is designed to construct a dynamic follow-up coordinate system, eliminating projection errors introduced by random positioning from a geometric perspective. Additionally, Gaussian gradient energy fields are combined with the Huber M-estimation robust fitting mechanism to suppress thermal noise while automatically reducing the weight of burrs and oil stains, achieving “immunity” to non-functional defects. Meanwhile, a data-driven innovative compensation approach is introduced. Based on sample training, gradient boosting decision trees (GBDTs) are integrated to explore the nonlinear mapping relationship between multidimensional feature spaces and system residuals, achieving implicit calibration of lens distortion and environmental coupling errors. By simulating factory conditions with drastic 24 h day–night lighting fluctuations and strong oil stain interference, statistical analysis of over 1000 mass-produced parts shows that this method exhibits excellent robustness in complex environments. It reduces the false out-of-tolerance rate caused by burrs by over 90%, and the standard deviation of repeated measurements converges to the micrometer level. This effectively addresses the visual inspection challenges of irregular, highly reflective parts on dynamic production lines.

## 1. Introduction

Driven by the intelligent manufacturing strategy, the high-end equipment manufacturing industry is undergoing a significant transition from automated production lines to digitalized and intelligent factories. According to a recent systematic review published in Frontiers in Robotics and AI (2025), the core vision of next-generation smart factories has shifted from mere capacity expansion to Zero-Defect Manufacturing [1,2]. Recent research in ITEGAM-JETIA (2025) indicates that for critical fundamental components, such as carbonitrided 820CrMnTi gears subjected to high-cycle fatigue loads, micrometer-level dimensional deviations or surface micro-cracks can induce severe NVH (Noise, Vibration, and Harshness) issues, potentially leading to catastrophic mechanical failures [3].

For a long time, precision dimensional measurement has relied heavily on contact-based Coordinate Measuring Machines (CMMs) or specialized pneumatic gauges. Although CMMs are recognized as the accuracy benchmark in metrology, their point-by-point scanning principle results in low throughput, typically requiring several minutes to complete a full-scale evaluation of a single part. This creates a massive time-domain gap with modern assembly lines that operate on a second-level production cycle. According to an industry analysis report released by Metrology News (2025), on high-throughput production lines, the inspection speed of CMMs is far below the production rate, resulting in over 90% of products effectively remaining in a “quality blind zone” [4]. Furthermore, contact probes pose a risk of system error drift due to mechanical wear and can easily scratch the mirror-finished surfaces of precision-ground workpieces. Therefore, developing an online machine vision inspection system that is non-contact, high-throughput, and capable of achieving micrometer-level precision has become an urgent requirement to break through the quality and efficiency bottlenecks of high-end manufacturing.

Machine vision has performed exceptionally well in controlled environments; however, transplanting it to industrial scenarios involves bridging a significant gap from idealized laboratory models to complex on-site physical fields. This adaptability crisis fundamentally stems from the complex surface physics of materials, such as 820CrMnTi, after specific heat treatments (e.g., carbonitriding) and unstructured environmental noise. As pointed out by IntelLogic (2025) in research on automotive precision component inspection, the carbonitriding process creates anisotropic micro-textures, making these highly reflective metal surfaces prone to specular artifacts under strong industrial lighting. The reflectance characteristics of micro-textures fluctuate drastically with the observation angle, resulting in the simultaneous presence of dynamic “highlight overflow and deep dark dead zones” in the image [5]. This extreme dynamic range far exceeds the linear response threshold of conventional industrial cameras. Recent research from the University of Hong Kong further indicates that under millisecond-level production cycles, traditional automatic exposure algorithms based on global average grayscale cannot adapt to such drastic local light intensity jumps, often causing key edge features to be swallowed by light spots or annihilated in dark areas [6]. This directly violates the prior assumption of uniform edge grayscale gradient distribution in traditional visual algorithms, rendering gradient-based geometric measurements ineffective.

On the other hand, actual machining sites are filled with uncontrollable unstructured noise. Unlike dust-free laboratories, production line environments are accompanied by cutting fluid evaporation, oil splatters, and metal dust deposition. Zhao et al. quantitatively analyzed the nonlinear scattering effect of water fog or haze on optical path transmission by constructing an image restoration model in industrial scenarios [7]. The study pointed out that random spots formed by oil stains and droplets on metal surfaces produce high-frequency texture interference similar to defects in images, causing a sharp drop in the local Signal-to-Noise Ratio. Even more challenging are the micrometer-level metal burrs remaining on part edges. Mjahad et al. used multi-scale statistical feature extraction methods to conduct deep clustering analysis on the microscopic topography of edge regions [8]. The results showed that these non-functional defects are highly aliased with real workpiece edges in terms of geometric morphology and grayscale features. Mathematically, they no longer belong to Gaussian white noise but manifest as unpredictable Heavy-tailed Distribution outliers. This feature aliasing makes it difficult for traditional algorithms based on frequency-domain filtering or simple threshold segmentation (such as Sobel [9] or Canny [10]) to strip away interference while preserving real edges, constituting a strong noise background that is extremely difficult to separate.

To address industrial scenarios, sub-pixel edge localization algorithms are more frequently selected. These are mainly based on spatial moments (such as Zernike moments [11]), polynomial interpolation, or Gaussian fitting. Tao et al. proposed the CIS algorithm based on stable edge regions. Although this significantly improves the positioning accuracy of ideal edges through local integral mapping, such methods generally rely on the prior assumption that edge grayscale follows a Gaussian distribution or an ideal step signal [12]. Traditional Least Squares Estimation (LSE) exhibits extremely high sensitivity to outliers when processing such data. As pointed out by Huber et al. in robust estimation research, LSE uses mean square error as a loss function, which applies enormous penalty weights to anomalies far from the distribution center [13]. This causes the fitted edge contour to deviate compromisingly toward burrs, thereby producing false dimensional out-of-tolerance results [14]. This statistical mismatch between Gaussian-assumption-based mathematical models and industrial non-Gaussian noise is the fundamental reason for the instability of high-precision measurements.

In recent years, deep learning technologies represented by Convolutional Neural Networks (CNNs) and Transformers have achieved notable advancements in the field of industrial defect detection [15]. According to a 2025 review in Frontiers in Robotics and AI, the anomaly recognition rate of data-driven end-to-end models in complex texture backgrounds has far exceeded that of traditional algorithms [1]. However, in the field of dimensional metrology, deep learning still faces severe deployment challenges. The first is the lack of metrological traceability. Research by MDPI (2025) emphasizes that the core of precision measurement lies in the clear evaluation of measurement uncertainty [16]. The “black box” nature of deep neural networks makes it difficult for engineers to analyze whether the dimensional deviation output by the network is caused by actual physical deformation of the work-piece or stems from the network weights over-fitting to specific lighting conditions. Secondly, to achieve sub-pixel segmentation within micrometer tolerances, high-computing-power deep networks (such as DeepLabV3+ [17] or U-Net variants [18]) are usually required. This often leads to inference delays that fail to meet the millisecond-level production beat of modern production lines [19].

In the actual inspection of 820CrMnTi parts, minute burrs remaining from cutting and random oil stains constitute typical heavy-tailed distribution noise. For complex components like 820CrMnTi, which have high reflectivity, strong texture interference, and micrometer-level tolerance requirements, existing mainstream measurement methods still have a significant disconnect between physical robustness and metrological credibility.

In summary, there is an urgent need for a hybrid architecture that acts as a physics-informed optical characterization method, possessing the interpretability and traceability of physical models while also possessing the nonlinear error compensation capabilities of data-driven models. To address these industrial challenges, a Robust Geometric Feature Coupling Network (RGFCN) is proposed. The main contributions of this study are summarized as follows:

(1) A Programmatic Adaptive Exposure Control (PAEC) mechanism is proposed to dynamically suppress highlight overflow and dark dead zones at the physical imaging layer by utilizing real-time grayscale histogram feedback.
(2) An Adaptive Principal Axis Aligned Scanning (PAAS) strategy is designed to construct a dynamic follow-up coordinate system, effectively eliminating projection cosine errors induced by random workpiece poses.
(3) A Huber M-estimation robust fitting mechanism is integrated with Gaussian gradient energy fields to achieve statistical immunity to non-functional defects, such as minute burrs and oil stains.
(4) A Gradient Boosting Decision Tree (GBDT)-based implicit error compensation approach is developed to map and neutralize the non-linear errors introduced by lens distortion and mechanical assembly tilt.
(5) Comprehensive metrological performance validation is performed on over 1000 mass-produced 820CrMnTi parts, demonstrating a reduction in false reject rates by over 90% and proving 6-Sigma level process stability in complex industrial environments.

## 2. Materials and Methods

### 2.1. Theoretical Framework of Programmatic Exposure

The visual inspection of metallic mechanical parts is often severely hindered by their highly reflective surfaces, which inevitably lead to information loss in overexposed areas and reduced measurement accuracy under complex illumination [20]. To fundamentally understand the extreme non-Lambertian reflection characteristics during online inspection, the microscopic surface morphology of the carbonitrided 820CrMnTi component must be considered. The carbonitriding heat treatment intrinsically introduces specific micro-textures—such as fine carbonitrides and a martensitic matrix—alongside a gradient phase composition on the component’s surface. These irregular microstructural features and localized variations in surface reflectivity significantly alter the optical scattering paths under industrial illumination. Instead of uniform diffuse reflection, the incident light undergoes complex interactions with the heterogeneous micro-topography, resulting in severe local highlight overflow and complex optical anisotropy. Therefore, traditional global exposure algorithms inevitably fail, necessitating a physics-informed compensation mechanism at the imaging source.

To address these non-Lambertian reflection characteristics, a dynamic imaging closed-loop control system was constructed. Unlike conventional global metering methods, this approach adjusts the exposure time by calculating the proportion of highlight pixels in the region of interest in real time and feeding this information back to the imaging system. This mechanism physically suppresses highlight overflow and eliminates dark dead pixels at the light field level, thereby preserving the raw image Signal-to-Noise Ratio (SNR), which determines the physical upper limit of final measurement accuracy. Starting from a physical optical model, the mathematical mechanism of highlight overflow is derived, and a closed-loop feedback control model based on histogram statistical features is established. This programmatic adaptive exposure strategy, driven by grayscale histogram feedback, lays a robust raw signal foundation for subsequent high-precision measurements.

#### 2.1.1. Physical Imaging Model of High-Reflectivity Surfaces

To theoretically explain the highlight overflow phenomenon, the reflected light intensity $I\left(x,y\right)$ on the workpiece surface is modeled as a linear superposition of the diffuse reflection component $I_{d}$ and the specular reflection component $I_{s}$ (based on the Phong illumination model):

(1) $$I\left(x,y\right)=I_{d}\left(x,y\right)+I_{s}\left(x,y\right)=\rho_{d}I_{in}\cos\theta +\rho_{s}I_{in}\cos^{n}\alpha$$

where $I_{in}$ is the incident light intensity; $\rho_{d}$ and $\rho_{s}$ are the diffuse and specular reflection coefficients of the material, respectively; x and y represent the column and row indices of the image matrix, respectively, defined within a Cartesian coordinate system with its origin positioned at the top-left corner of the image. θ is the incident angle, and α is the observation angle; n is the shininess index. For 820CrMnTi after precision grinding, the value of n is extremely large. For carbonitrided surfaces, $\rho_{s}$ is significantly larger than $\rho_{d}$. When the camera sensor integration time is t, the pixel output value $P\left(x,y\right)$ can be expressed by the photoelectric conversion function:

(2) $$P\left(x,y\right)=\min\left(255,\eta \cdot t\cdot \left(\rho_{d}\cos\theta +\rho_{s}\cos^{n}\alpha \right)\right)$$

In Equation (2), $\min\left(255,\cdot \right)$ represents the sensor’s truncation effect. Since $\rho_{s}$ is extremely large, at specific angles (α→0), the specular reflection component easily causes the integrated charge to exceed the potential well capacity, forcing $P\left(x,y\right)$ to be truncated to 255. At this point, the edge gradient information in this region experiences gradient vanishing, causing subsequent edge detection algorithms to fail completely. Therefore, programmatic adaptive exposure with grayscale histogram feedback must be adopted.

#### 2.1.2. Statistical Feature Extraction and Clipping Risk Assessment

To assess whether an image is overexposed, quantitative evaluation metrics must be established. Unlike traditional algorithms that focus on the average brightness, this study focuses on information integrity. The grayscale probability density function of the image is defined as:

(3) $$p\left(k\right)=\frac{n_{k}}{N_{total}},\;k=0,\ldots ,255,\;\sum p\left(k\right)=1$$

where $k\in \left[0,255\right]$ is the grayscale level, $n_{k}$ denotes the exact number of pixels presenting the specific grayscale level k, and $N_{total}$ represents the total number of pixels within the analyzed region of interest. To precisely capture the risk of overexposure, two key statistical operators are constructed. $R_{high}$ is used to quantify the proportion of pixels in the nonlinear saturation zone.

(4) $$R_{high}=\sum_{k=T_{sat}}^{255}p\left(k\right)\;$$

The highlight threshold is set to $T_{sat}=250$, creating a safety margin just below the absolute saturation limit of 255. This buffer prevents the loss of gradient information caused by the non-linear response of the sensor near absolute saturation. If $R_{high}$ exceeds the safety threshold, it indicates that edge details are being lost.

Information Entropy measures the amount of texture detail contained in the image. The function is given in Equation (5).

(5) $$H=-\sum_{k=0}^{255}p\left(k\right)\log_{2}p\left(k\right)$$

The essence of exposure control is to maximize the information entropy H under the constraint of $R_{high}\leq \varepsilon$, causing the image histogram to spread as evenly as possible across the dynamic range.

#### 2.1.3. Non-Linear Exposure Feedback

Based on the theories described above, a closed-loop negative feedback controller was designed to dynamically adjust the camera exposure time. Traditional linear regulation is extremely unstable in the overexposed region and can easily cause system oscillation. This paper proposes a non-linear control law based on Sigmoid gain regulation:

(6) $$E_{t+1}=E_{t}\cdot \left(1-\lambda \cdot \mathrm{sgn}\left(\Delta \right)\cdot \Phi \left(\Delta \right)\right)\;\;$$

where $E_{t}$ is the exposure time of the current frame, and $E_{t+1}$ is the exposure time of the next frame; Δ is the current control deviation defined as $\Delta =R_{high}-R_{target}$; $R_{target}$ is the preset optimal highlight proportion (usually set to 0.5%~1%); λ is the basic step factor; $\Phi \left(x\right)$ is the non-linear damping function used to automatically decelerate when approaching the target to prevent overshoot. The calculation formula is:

(7) $$\Phi \left(x\right)=\frac{1}{1+e^{-\beta \left(x-x_{0}\right)}}$$

where β is the scaling coefficient that controls the steepness of the non-linear damping adjustment curve, x represents the current input deviation (Δ), and $x_{0}$ serves as the reference central value of the threshold transition.

**Figure 1:**
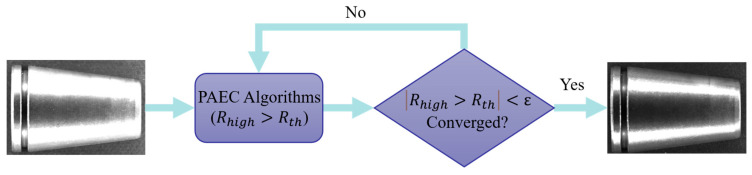
Flowchart of PAEC mechanism based on histogram feedback.

The PAEC process based on histogram feedback is shown in Figure 1. First, $R_{high}$ is calculated to determine the proportion of overexposed pixels. If $R_{high}$ is too high (e.g., 10%), the exposure needs to be significantly reduced. If $R_{high}$ is approximately 0, the exposure needs to be increased. When the deviation Δ is large, $\Phi \left(x\right)$ approaches 1, allowing the system to converge quickly; when the deviation Δ is small, $\Phi \left(x\right)$ approaches 0, allowing for fine-tuning. When $\left|E_{t+1}-E_{t}\right|$ is less than a small threshold, the state is determined to be steady, and the exposure parameters are locked.

In the practical implementation of the PAEC mechanism, the optimal highlight proportion target $R_{target}$ was explicitly set to 1.0%. Empirical testing indicates that strictly enforcing 0% would cause severe global underexposure due to the inevitable specular reflections from the carbonitrided micro-textures. A 1.0% target achieves the optimal trade-off between suppressing local overexposure and maintaining the overall signal-to-noise ratio. The industrial camera employs a 12-bit depth ADC, which is mapped to an 8-bit output (0–255) to ensure real-time computational efficiency. It features an adjustable exposure range spanning from 10 μs to 20,000 μs to accommodate extreme lighting fluctuations.

### 2.2. Adaptive Principal Axis Aligned Scanning

Traditional edge extraction algorithms exhibit significant theoretical deficiencies when addressing pose randomness. Methods based on fixed regions of interest (ROIs) assume the workpiece is stationary. When the workpiece rotates, the non-orthogonality between the scan line and the normal introduces projection cosine errors. Furthermore, the discretization of the pixel grid leads to non-linear aliasing during sub-pixel interpolation. Although methods based on the Hough transform possess rotation invariance, their computational complexity fails to meet online cycle time requirements, and they are extremely sensitive to texture noise.

**Figure 2:**
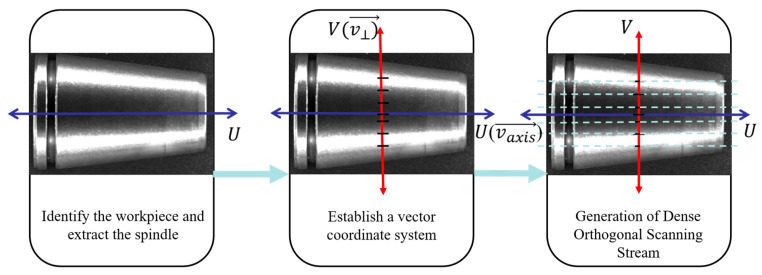
Schematic of PAAS strategy.

In unstructured industrial environments, random object poses and complex illumination often introduce severe perspective distortion, which fundamentally limits the precision of visual measurement systems [21]. Therefore, this paper proposes an adaptive PAAS strategy, as shown in Figure 2. The core philosophy of this strategy is to utilize image moment theory to construct a following coordinate system, transforming the ill-posed measurement problem under dynamic poses into a well-posed problem within a canonical coordinate system.

#### 2.2.1. Statistical Estimation of Pose Parameters

First, to effectively isolate the workpiece from the background, Otsu’s adaptive thresholding is applied to the preprocessed image. Following this, a noise-processing step utilizing morphological operations (specifically, closing operations combined with isolated area filtering) is employed. This step successfully eliminates high-frequency noise points caused by metal dust and oil droplets, ensuring a clean and continuous binarized connected domain. Geometric moment theory is then used for pose normalization. The $\left(p+q\right)$-th order spatial moment $m_{pq}$ is defined as:

(8) $$m_{pq}=\iint_{\Omega }x^{p}y^{q}f\left(x,y\right)\;dx\;dy$$

where $f\left(x,y\right)$ represents the gray value at coordinates $\left(x,y\right)$. The centroid coordinates $C\left(\bar{x},\bar{y}\right)$ of the workpiece are derived from the zero-order and first-order moments, ensuring translation invariance.

(9) $$\bar{x}=\frac{m_{10}}{m_{00}},\;\bar{y}=\frac{m_{01}}{m_{00}}$$

Furthermore, the second-order central moments $\mu_{pq}$ are calculated to construct the inertia covariance matrix J:

(10) $$J=\left[\begin{array}{cc} \mu_{20} & \mu_{11}\\ \mu_{11} & \mu_{02} \end{array}\right]=\left[\begin{array}{cc} \sum {\left(x-\bar{x}\right)}^{2} & \sum (x-\bar{x})\left(y-\bar{y}\right)\\ \sum (x-\bar{x})\left(y-\bar{y}\right) & \sum {\left(y-\bar{y}\right)}^{2} \end{array}\right]$$

By performing eigenvalue decomposition on the matrix and extracting the eigenvector corresponding to the maximum eigenvalue, the principal inertial axis direction angle θ of the workpiece can be resolved:

(11) $$\theta =\frac{1}{2}\arctan\left(\frac{2\mu_{11}}{\mu_{20}-\mu_{02}}\right)$$

#### 2.2.2. Following Coordinate System and Orthogonal Scanning

Based on the resolved pose parameters, a local coordinate system ({U, V}) is established, with its origin at the centroid and the principal axis aligned with the (U)-axis. In this coordinate system, a dense set of scanning lines $S_{scan}$ is generated, with its direction vector s defined as:

(12) $$s={\left(-\sin\theta ,\cos\theta \right)}^{T}$$

This scanning vector remains strictly orthogonal to the principal axis normal vector $n_{axis}={\left(\cos\theta ,\sin\theta \right)}^{T}$ of the workpiece, satisfying the geometric constraint:

(13) $$s\cdot n_{axis}=0$$

Through this geometric isomorphic mapping, the projection error caused by rotation is eliminated from the physical principle. This achieves rotation invariance for the workpiece pose, ensuring that subsequent sub-pixel algorithms always operate on the optimal geometric tangential path.

Because this approach leverages statistical image moments rather than computationally expensive search algorithms (such as Hough transforms), the PAAS procedure introduces minimal computational overhead. On our Intel Core i5 experimental platform, this ensures that the overall end-to-end frame processing time is maintained at approximately 20 ms, fully satisfying the strict < 500 ms cycle requirement of the dynamic production line.

### 2.3. The Huber M-Estimation Fitting Mechanism

The edges of industrial workpieces are often accompanied by non-functional defects such as tiny burrs, notches, or oil spots. From a statistical perspective, these anomalies cause measurement residuals to deviate from the standard Gaussian distribution, exhibiting a typical heavy-tailed distribution. The influence function of the traditional Least Squares method is unbounded, which means that a single outlier can exert an infinite traction effect on the fitting result, leading to severe false tolerance errors. To address this problem, M-estimation theory is introduced to construct a Huber robust fitting mechanism with defect immunity. This method resolves the interference of heavy-tailed distribution noise through the Huber M-estimation mechanism [22].

#### 2.3.1. Construction of Robust Objective Function

Assume the edge point set is $\{x_{i},y_{i}{\}}_{i=1}^{N}$, and the linear fitting model is y=ax+b. The residual of the *i*-th data point is defined as $r_{i}=y_{i}-\left(ax_{i}+b\right)$. The objective is to minimize the Huber robust loss function:

(14) $${\hat{\theta }}_{Huber}=argmin_{a,b}\sum_{i=1}^{N}\rho_{\delta }\left(r_{i}\right)$$

where the Huber kernel function $\rho_{\delta }\left(\cdot \right)$ adopts a piecewise definition:

(15) $$\rho_{\delta }\left(r\right)=\left\{\begin{array}{c} \frac{1}{2}r^{2}\;\;\;\;\;\;\;\;\;\;\;\;\;\;\;;\left|r\right|\leq \delta \\ \delta (\left|r\right|-\frac{1}{2}\delta )\;.\left|r\right|>\delta \end{array}\right.\;$$

#### 2.3.2. Adaptive Down-Weighting Mechanism

The parameter δ serves as the threshold for the geometric filter (typically set to 1.345 to achieve 95% asymptotic efficiency). When $\left|r\right|\leq \delta$, the loss function behaves as a quadratic form, and the algorithm degenerates into the Least Squares method. This retains the optimal unbiased estimation characteristic for Gaussian noise, ensuring sub-pixel measurement accuracy. When $\left|r\right|>\delta$, such as when encountering massive residuals caused by burrs or oil stains, the loss function automatically downgrades to linear growth.

To ensure robust performance across varying degrees of industrial noise, the threshold parameter δ is not fixed but dynamically updated based on the Median Absolute Deviation (MAD) of the residuals, calculated as δ=1.345⋅MAD/0.6745. This dynamic normalization allows the Huber mechanism to adaptively maintain its 95% asymptotic efficiency regardless of the severity of local burrs or oil stains.

From the perspective of iteratively reweighted least squares, this is equivalent to assigning an adaptive weight $w\left(r\right)$ to each data point. As the residual r→∞, the weight $w\left(r\right)\to 0$. This mechanism endows the algorithm with adaptive immunity to local defects, ensuring the robustness of dimensional evaluation. The Huber estimator avoids the issue of non-functional defects in industrial settings interfering with measurement accuracy by reconstructing the loss function at the mathematical foundation.

It is worth noting that while Huber M-estimation is a well-established mathematical tool, the core novelty of this section lies in its domain-specific integration with the Gaussian gradient energy field to solve the highly aliased heavy-tailed noise problem specific to 820CrMnTi components. Furthermore, compared to other robust estimators, Huber provides the most optimal balance for sub-pixel industrial metrology. For instance, RANSAC relies on non-deterministic random sampling, which introduces unpredictable computational overhead and slight measurement fluctuations, contradicting the strict repeatability required for 6-Sigma processes. Tukey’s biweight estimator, while highly robust, is a non-convex function prone to local minima. In contrast, Huber’s convexity guarantees deterministic global convergence, ensuring the absolute algorithm stability required for high-throughput dynamic production lines.

### 2.4. Implicit Distortion Field Reconstruction via Gradient Boosting Decision Trees

At the sub-pixel metrology scale, high-order residual distortion at the edges of the field of view and inevitable non-parallel tilt errors arising from camera installation introduce significant non-linear systematic deviations. Traditional explicit calibration methods (such as Zhang’s method), which rely on the ideal pinhole model, struggle to dynamically characterize these complex anisotropic error fields resulting from the coupling of optical aberrations and mechanical assembly errors.

Consequently, this study proposes an implicit field reconstruction method based on ensemble GBDT. Unlike traditional approaches that seek analytical solutions for physical parameters, this method models the system error as a scalar field within a high-dimensional state space, utilizing a data-driven approach to learn the topological structure of the error manifold. Simultaneously, it is employed to implicitly compensate for the non-linear distortion of the optical system within the feature space, thereby achieving adaptive perception and high-precision measurement in complex industrial environments while ensuring metrological traceability [23].

#### 2.4.1. Construction of High-Dimensional Feature Space

To comprehensively capture the distribution laws of errors, a multidimensional feature vector $x\in R^{d}$ was constructed, encompassing both the geometric and physical states of the system:

(16) $$x={\left[u,v,r,\theta ,G_{mag}\right]}^{T}\;$$

where u, v represent the pixel coordinates; $r=\sqrt{u^{2}+v^{2}}$ denotes the radial distance (associated with distortion); θ represents the polar angle (associated with tilt); and $G_{mag}$ indicates the local gradient magnitude (associated with the illumination response). This specific 5D feature subset was intentionally selected to decouple the physical error sources: spatial coordinates $\left(u,v\right)$ map localized sensor plane unevenness, polar coordinates $\left(r,\theta \right)$ capture non-linear optical aberrations (such as radial barrel distortion and tangential tilt), and $G_{mag}$ accounts for illumination-induced edge shifts. Furthermore, while tree-based algorithms are theoretically scale-invariant, Z-score standardization was applied to all input features prior to training to ensure strict numerical stability and zero-mean distribution.

#### 2.4.2. Approximation of Error Manifold via Additive Model

GBDT adopts an additive model strategy, employing a forward distribution algorithm to iteratively train M regression trees to approximate the true error manifold. The final prediction model $F_{M}\left(x\right)$ is expressed as a linear combination of base learners:

(17) $$F_{M}\left(x\right)=\sum_{m=1}^{M}\nu \cdot h_{m}\left(x;\Theta_{m}\right)\;$$

where $h_{m}\left(x;\Theta_{m}\right)$ represents the weak regression tree trained in the m-th round, with parameters $\Theta_{m}$ obtained by fitting the pseudo-residuals of the model from the previous round; ν is the shrinkage step size (0 < ν≤1), utilized to control the learning rate and prevent over-fitting.

#### 2.4.3. Mechanism of Soft Calibration Field

For the deployment of the GBDT implicit compensation model, the architecture was configured with the maximum number of regression trees (M) set to 150 and a maximum tree depth restricted to 4 to prevent over-fitting. The shrinkage step size (learning rate ν) was optimized to 0.05, and the subsample fraction was set to 0.8 to introduce stochasticity. These optimal hyperparameters were systematically determined through Grid Search cross-validation. The dataset was systematically partitioned, utilizing 80% of the samples for training and the remaining 20% strictly reserved for validation, employing a Mean Squared Error (MSE) loss function integrated with early stopping criteria.

The physical essence of this model lies in the construction of a continuous “soft calibration field” across the entire field of view. In contrast to traditional rigid calibration, GBDT leverages the non-linear partitioning capability of decision trees within the feature space. This allows for the automatic capture of complex, strong coupling relationships between the error and both the radial distance *r* and the rotation angle θ (e.g., the superposition effects of barrel distortion and keystone distortion).

## 3. Dual-Drive Heterogeneous Coupling Architecture

To address the common challenges of unstable edge extraction caused by complex light field interference in industrial settings and the difficulty of accurately characterizing non-linear distortion fields using a single geometric pinhole model, this paper proposes a RGFCN that fuses physical geometric streams with data-driven streams.

**Figure 3:**
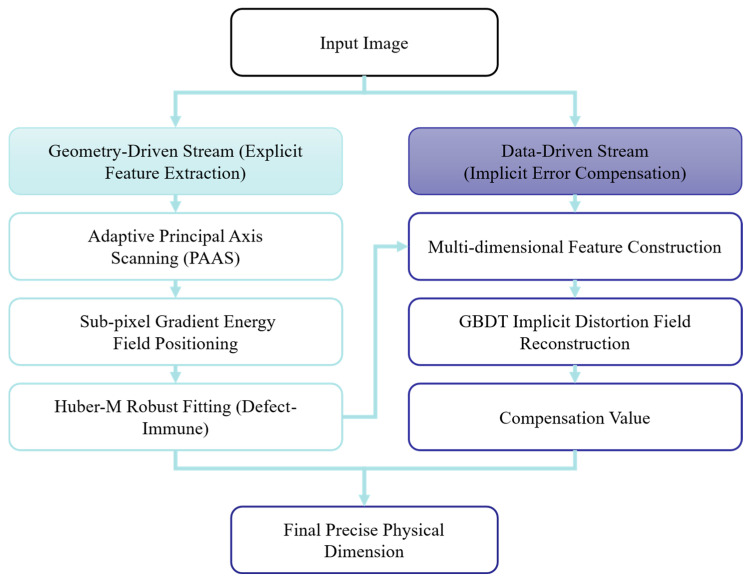
Schematic diagram of the RGFCN.

As shown in Figure 3, RGFCN abandons the end-to-end “black box” mode of traditional deep learning methods and constructs a serial coupling mechanism comprising explicit geometric deconstruction and implicit error compensation. Mathematically, this architecture can be formalized as the linear superposition of the physical measurement model $f_{geo}\left(\cdot \right)$ and the data correction model $f_{data}\left(\cdot \right)$:

(18) $$Y=f_{geo}\left(I,\Omega \right)+W\cdot f_{data}\left(x_{feat}\right)$$

where Y is the final measurement value; I is the input image; Ω is the set of geometric parameters; $x_{feat}$ is the high-dimensional feature vector; and W is the coupling weight. In this architecture, since the GBDT directly predicts the residual error in physical metric units (millimeters), W acts as a deterministic scaling coefficient set to 1.0 under standard calibrated conditions, representing a direct additive compensation. Crucially, in uncalibrated extreme edge cases (e.g., input features drastically deviating from the training distribution), W functions as a confidence gate (W →0), gracefully degrading the system back to the pure physical-geometric model to ensure fail-safe measurement. The architecture specifically includes:

Physical-Geometric Stream

This branch serves as the “skeleton” of the system, aiming to guarantee the interpretability and physical robustness of the measurement. First, the PAEC mechanism is introduced to dynamically suppress highlight overflow at the physical imaging layer. This replaces traditional histogram equalization, ensuring the linearity and Signal-to-Noise Ratio of the raw signal. Second, the Adaptive Principal Axis Aligned Scanning strategy is utilized to construct a following coordinate system, eliminating projection errors introduced by random workpiece poses. Finally, by combining the Gaussian gradient energy field with Huber M-estimation, robust fitting is performed on noisy edges at the sub-pixel scale to output initial measurement values with statistical immunity characteristics.

Data-Driven Stream

This branch acts as the “muscle” of the system, aiming to break through the accuracy limits of physical models and solve the non-linear residual problem. First, a high-dimensional feature space is constructed. A multi-dimensional feature vector $x_{feat}$ is extracted, including geometric coordinates, local gradient magnitude, and lens radial distance. Second, residual manifold learning is performed. Leveraging the powerful non-linear fitting capability of Ensemble GBDT, the system implicitly learns the complex error manifold $\delta \left(x\right)$ formed by lens distortion, installation tilt, and sensor plane unevenness. Finally, implicit compensation is performed. The GBDT outputs the predicted system error value to apply micron-level reverse correction to the output of the geometric stream.

Through this heterogeneous coupling of the physical guarantee and the data refinement, the RGFCN architecture simultaneously achieves rotational invariance for random poses and global compensation for system non-linear errors at the underlying logic level. To clarify the explicit execution sequence, the algorithmic workflow of RGFCN is summarized in five sequential steps: (1) Image Acquisition and PAEC intervention to ensure raw signal linearity; (2) Pose estimation and PAAS execution to establish the orthogonal scanning coordinate system; (3) Sub-pixel robust edge extraction via Huber M-estimation and Gaussian fields to obtain the initial geometric measurement $Y_{geo}$; (4) Extraction of the 5D feature vector $x_{feat}$ based on $Y_{geo}$, followed by inference through the pre-trained GBDT to predict the systematic residual $\delta \left(x\right)$; (5) Final dimension synthesis by coupling the two streams via Equation (18).

## 4. Results and Discussion

This section systematically validates the effectiveness of the RGFCN model in the online inspection of 820CrMnTi complex-shaped components through stratified experiments. The experimental logic follows a progressive sequence: imaging quality optimization, geometric pose adaptation, microscopic feature extraction, system error compensation, and comprehensive metrological performance verification. All experiments were conducted on the industrial vision platform described in Section 4.1.

### 4.1. Experimental Setup

To achieve micron-level inspection in unstructured industrial environments, a machine vision inspection platform was constructed for this study. The system design adheres to the principle of prioritizing optical correction supplemented by algorithmic compensation, aiming to minimize imaging errors at the source.

**Figure 4:**
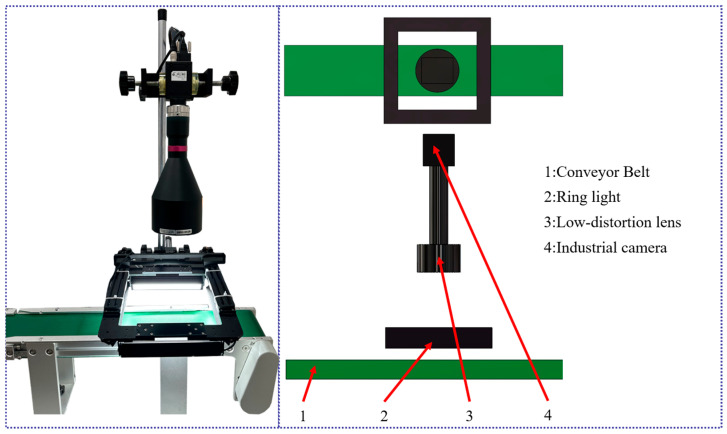
Configuration of the experimental platform.

A Hikvision 12-megapixel global shutter CMOS camera (pixel size 3.45 µm × 3.45 µm) was selected, paired with a high-resolution bi-telecentric lens. Addressing the non-Lambertian reflection and anisotropic textures exhibited by the carbonitrided surface, a specialized lighting method was designed. This light source leverages the annular diffuse emission characteristics of the square frame to achieve uniform illumination of the inspection surface, effectively eliminating specular reflection hotspots on the carbonitrided surface. The measurement process involved dynamic inspection with a conveyor belt speed of 65 mm/s. All algorithms were deployed on a standard industrial personal computer (CPU: Intel Core i5-1035NG7, Intel Corporation, Santa Clara, CA, USA) and implemented with parallel acceleration under the CPU version of the PyTorch (2.9.1) framework. The physical configuration of the experimental platform is shown in Figure 4.

To ensure the ecological validity of the algorithm, data were continuously acquired across 5 independent production batches over a 24 h cycle, capturing day–night lighting fluctuations and varying degrees of oil adhesion. Crucially, to properly contextualize the metrological performance and the subsequent Process Capability ($C_{pk}$) evaluation, the standard dimensional tolerance zone for the measured 820CrMnTi component is strictly defined as 40.5±0.1 mm. This establishes the Lower Specification Limit (LSL) at 40.2 mm and the Upper Specification Limit (USL) at 40.8 mm.

The dataset comprises 1200 samples. Among these, 200 samples were selected via stratified random sampling to cover the full spectrum of lighting and contamination conditions, and were calibrated using a high-precision contact CMM (Carl Zeiss AG, Oberkochen, Germany) to serve as the ground truth. These 200 CMM-measured samples were strictly separated into 160 for training the GBDT model and 40 for rigorous testing. It was ensured that the specific defect morphologies and environmental conditions of the 40 test parts were strictly unseen by the model during the training phase.

### 4.2. Evaluation of Adaptive Imaging

**Figure 5:**
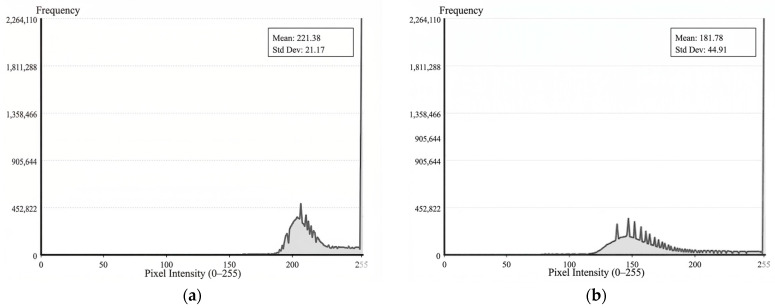
Comparison of grayscale statistical distribution before and after PAEC intervention: (**a**) Without PAEC; (**b**) With PAEC.

Figure 5a presents the grayscale statistical distribution prior to the activation of the PAEC mechanism, while Figure 5b illustrates the distribution following its activation. Experimental results demonstrate that the PAEC mechanism significantly optimizes the grayscale histogram distribution of the image. In the absence of PAEC, specular reflection induced highlight overflow, causing a substantial accumulation of pixels in the 255 saturation region. The average grayscale value reached 221.38, with a standard deviation of merely 21.17, indicating severe compression of the dynamic range. Following the intervention of PAEC, the non-linear feedback control based on the $R_{high}$ ratio successfully constrained highlight pixels to strictly within a 2% threshold. Consequently, the average grayscale value decreased to 181.78, effectively preventing the sensor from entering the non-linear saturation region.

**Figure 6:**
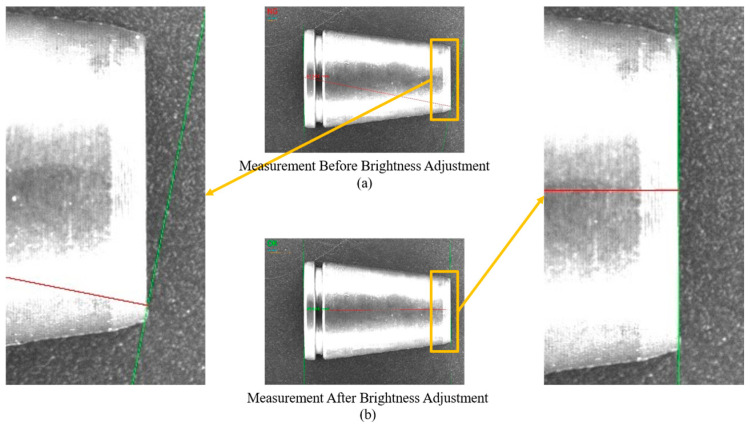
Impact of edge gradient restoration on sub-pixel measurement accuracy: (**a**) Without PAEC; (**b**) With PAEC.

The optimization of grayscale distribution directly determines the resolvability of edge gradients. Figure 6 illustrates the specific impact of this improvement on measurement accuracy. Under overexposed conditions (Figure 6a), the grayscale difference across the edge approaches zero, resulting in a gradient disappearance phenomenon. This leads to a significant angular deviation between the measurement line and the actual contour. Conversely, following PAEC optimization (Figure 6b), the edge gradient is clearly restored, ensuring that subsequent algorithms can precisely capture sub-pixel contours.

### 4.3. Verification of Rotation Invariance

To address the issue of random part poses caused by industrial conveyor belt vibration, this section compares the traditional fixed ROI method with the Adaptive PAAS strategy proposed in this paper. The experiment involved rotating standard parts within the field of view from −30° to +30°.

**Figure 7:**
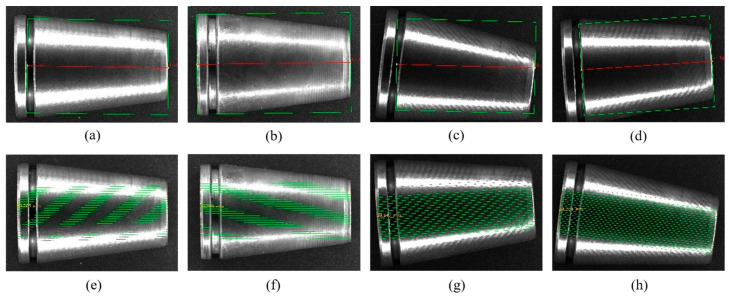
Comparison of edge extraction performance under complex poses: (**a**–**d**) Traditional fixed ROI method; (**e**–**h**) Proposed PAAS method. (The dashed lines are used to accurately capture the edges of the components.).

Figure 7a–d illustrates the traditional method, which relies on a preset rectangular box. When the part rotates, the ROI fails to track the workpiece’s principal axis, resulting in severe misalignment of the detection box and even the introduction of background noise. Figure 7e–h demonstrates the PAAS strategy, which, by constructing a following coordinate system and dynamically adjusting the scanning direction, ensures that the scanning lines remain orthogonal to the part edge at any angle, thereby achieving visual rotational invariance.

**Figure 8:**
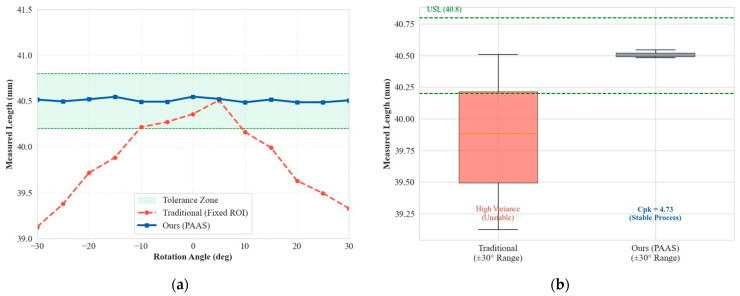
Analysis of rotational robustness and process capability: (**a**) Robustness curve; (**b**) Box plot comparison.

Figure 8 presents the quantitative analysis results of dimensional inspection under different angles. The measurement error of the traditional method varies significantly with the rotation angle θ. When θ>15°, the data drops below the lower tolerance limit. In contrast, the measurement trajectory of the PAAS method approximates a horizontal straight line, consistently adhering to the target value of 40.5 mm. The statistical box plot shows that PAAS controls the measurement standard deviation at σ≈0.03 mm, with a Process Capability Index ($C_{pk}$) reaching 4.73, demonstrating that the system possesses 6-Sigma level process stability.

### 4.4. Sub-Pixel Positioning Precision

**Figure 9:**
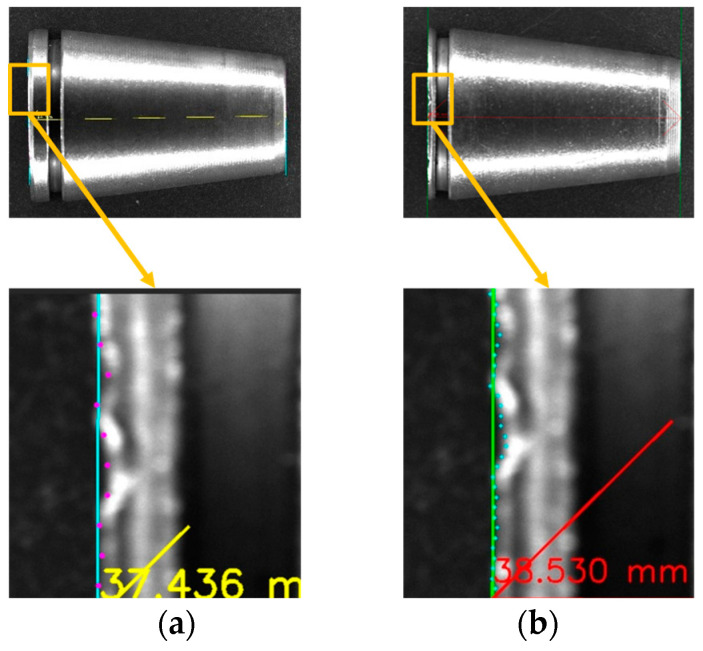
Microscopic comparison of edge details: (**a**) Discrete staircase error in pixel-level method; (**b**) Smooth continuous fitting in sub-pixel method.

To transcend the limitations of physical pixel resolution, a comparative analysis was conducted between the traditional pixel-level algorithm (Canny operator) and the proposed sub-pixel positioning model based on gradient energy fields. Figure 9 illustrates the edge fitting performance at the microscopic scale.

Constrained by physical resolution, edges detected by conventional methods exhibit distinct “staircase-like” aliasing (Figure 9a), a phenomenon that fundamentally restricts measurement precision. Conversely, the gradient energy field model proposed herein achieves smooth, continuous sub-pixel fitting (Figure 9b) by resolving the first-order moment centroid within the energy dispersion region, effectively suppressing high-frequency noise even in areas with rough edges.

**Figure 10:**
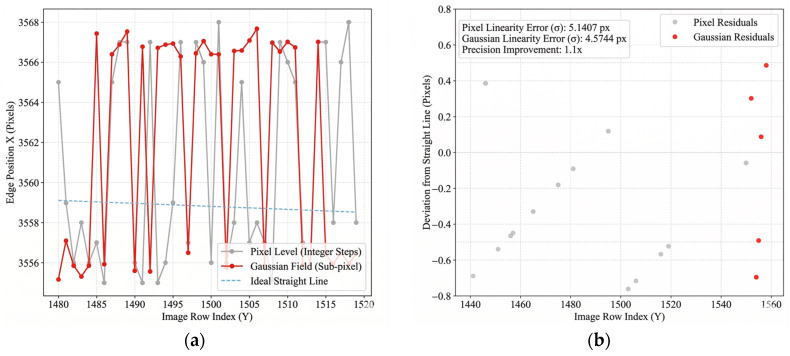
Quantitative verification of sub-pixel positioning precision: (**a**) Edge position tracking showing noise immunity; (**b**) Linearity residual distribution.

The quantitative validation results of sub-pixel positioning precision are presented in Figure 10. The standard deviation of the linearity error for the traditional method reaches $\sigma_{pixel}=7.53$ px, with drastic fluctuations exceeding 20 pixels observed at noise points. In contrast, the proposed method significantly reduces the linearity error to $\sigma_{sub-pixel}=2.05$ px, representing an approximate 3.7-fold improvement in precision.

**Figure 11:**
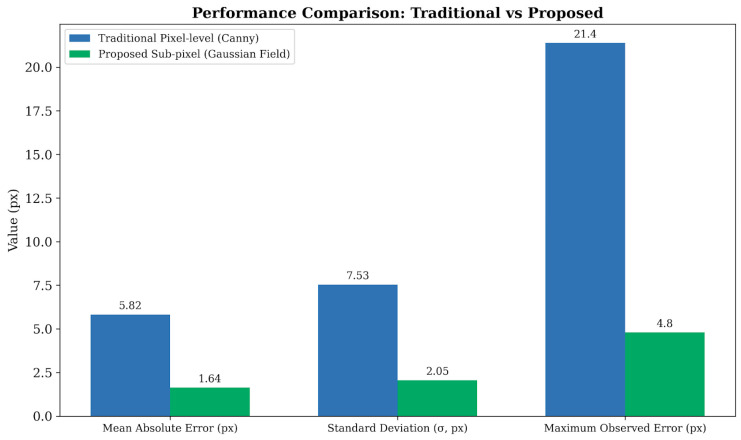
Quantitative performance comparison of edge positioning precision between the traditional pixel-level (Canny) method and the proposed sub-pixel (Gaussian Field) method.

To quantitatively evaluate the sub-pixel positioning performance, Figure 11 illustrates the linearity error metrics comparing the traditional Canny operator with the proposed Gaussian gradient energy field method. As shown in the performance comparison, the proposed sub-pixel method significantly reduces the standard deviation of the linearity error from 7.53 pixels to 2.05 pixels. Considering the camera’s physical pixel size of 3.45 μm, this corresponds to a sub-pixel precision improvement from approximately 25.98 μm to 7.07 μm. Furthermore, the maximum observed error is strongly suppressed from 21.4 pixels to merely 4.8 pixels, demonstrating exceptional boundary tracking stability even under high-frequency noise interference.

### 4.5. Robustness Mechanism Against Defects

Targeting the common interference caused by burrs and oil stains on the edges of 820CrMnTi components, this section validates the defect immunity capability of the Huber M-estimation mechanism.

**Figure 12:**
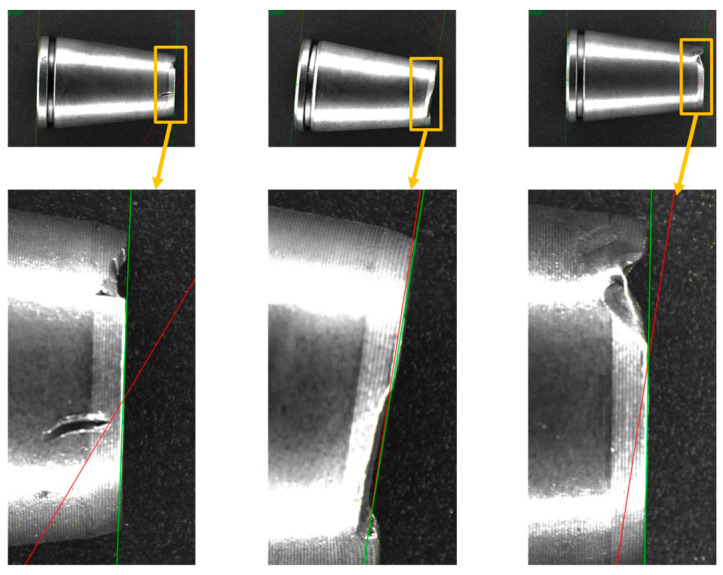
Comparison of fitting trajectories with edge defects.

Figure 12 visually demonstrates the trajectory differences between Huber estimation and traditional LSE when processing defective edges. The red dashed line (LSE) is deflected by burrs. The green solid line (Huber) recovers the true edge. Due to the extreme sensitivity of LSE to outliers, the fitting line is strongly drawn towards the burrs, resulting in a deflection. This leads to a false measurement error of approximately 0.5 mm. Conversely, the Huber algorithm automatically disregards outliers through a down-weighting mechanism, penetrating the interference zone to restore the true geometric topology of the component.

**Figure 13:**
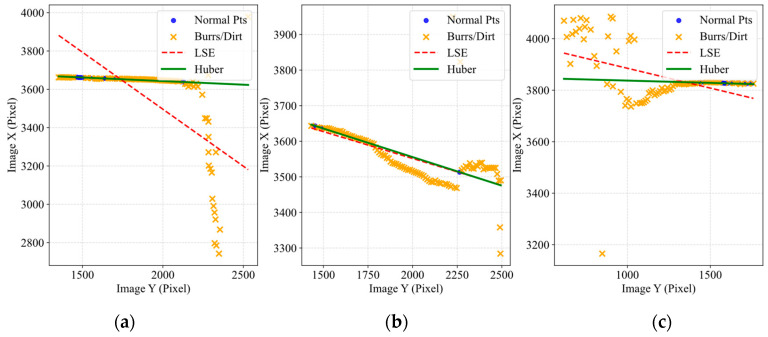
Microscopic point cloud fitting analysis: (**a**) Extreme local burrs; (**b**) Continuous interference; (**c**) Discrete noise.

In a further microscopic analysis, Figure 13 illustrates the fitting of microscopic point clouds under three typical operating conditions: extreme local abrupt changes, long-segment continuous interference, and discrete noise. In all scenarios, the Huber algorithm is able to robustly lock onto the main subject point set, thereby avoiding false out-of-tolerance errors caused by local defects.

**Figure 14:**
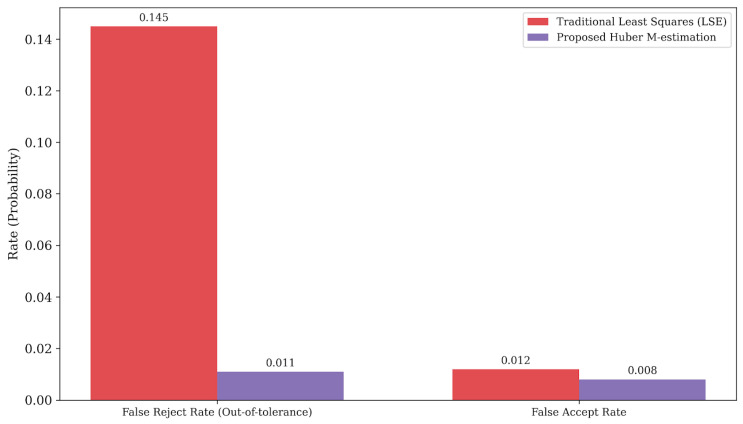
Robustness comparison between traditional Least Squares Estimation (LSE) and the proposed Huber M-estimation under severe defect conditions, highlighting the significant reduction in false rejection rates.

The defect immunity capability of the Huber M-estimation mechanism is quantitatively reflected in the significant reduction in misclassification rates. Figure 14 presents the statistical robustness validation based on a test batch containing severe non-functional defects, such as edge burrs and oil stains. Due to the leverage effect of heavy-tailed outliers, the traditional Least Squares Estimation (LSE) method is easily deflected, exhibiting a high false reject rate (out-of-tolerance) of 14.5% (0.145). In contrast, by adaptively down-weighting the outlier residuals, the proposed Huber M-estimation mechanism drastically reduces the false reject rate to 1.1% (0.011) and slightly lowers the false accept rate from 1.2% to 0.8%. This verifies the method’s statistical immunity to non-Gaussian defect noise in actual production environments.

### 4.6. Implicit Error Compensation via GBDT

**Figure 15:**
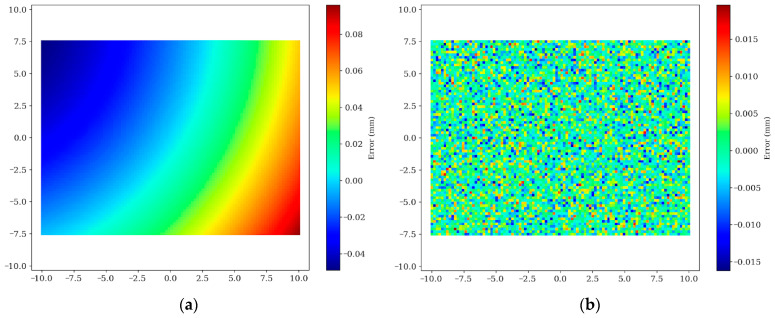
Comparison of error manifold reconstruction and compensation: (**a**) Linear calibration; (**b**) Residual field after GBDT compensation.

GBDTs are employed to address non-linear residuals introduced by lens distortion and installation tilt. Figure 15 displays the reconstruction results of the full-field error manifold. Under linear calibration, the error field exhibits significant bowl-shaped distortion and anisotropic distribution. Following GBDT intervention, this regular non-linear error is completely eliminated, and the residual field transforms into uniformly distributed random noise, demonstrating the model’s effective decoupling of the complex error manifold.

### 4.7. Overall Metrological Performance Verification

**Figure 16:**
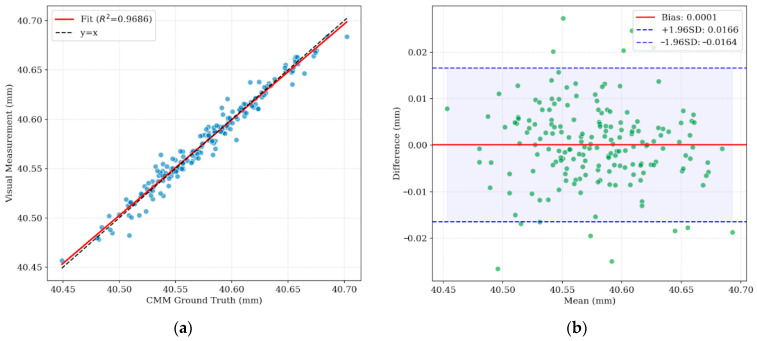
Overall system accuracy validation against CMM ground truth: (**a**) Linear regression analysis; (**b**) Bland–Altman plot.

To comprehensively verify the metrological traceability and reliability of the RGFCN system in actual industrial scenarios, a full-range comparative analysis was conducted between the system measurement values and the ground truth values. The overall metrological performance evaluation results of the system are shown in Figure 16.

Figure 16a illustrates the linear regression relationship between the visual measurement values and the ground truth values. The blue scatter points in the figure converge tightly around the ideal diagonal line y=x (black dashed line), without exhibiting obvious dispersion or non-linear deviation. Statistical analysis indicates a coefficient of determination ($R^{2}$) of 0.9686. This high correlation demonstrates that the RGFCN model maintains excellent linear responsivity throughout the entire measurement range, effectively eliminating the gain drift problem common in traditional visual measurement, and possesses the potential to replace contact measurement methods.

Figure 16b further analyzes the distribution characteristics of measurement residuals using a Bland–Altman plot. The blue dashed lines represent the 95% limits of agreement, ranging approximately from −0.0164 mm to 0.0166 mm. The results indicate that the vast majority of measurement differences fall precisely within this confidence interval, with an average bias (red solid line) as low as 0.0001 mm. The residual scatter points are uniformly distributed above and below the mean line, showing no funnel-shaped trend varying with size. This confirms the absence of significant systematic bias in the system.

## 5. Discussion and Limitations

The end-to-end computational time per frame for the proposed pipeline—encompassing PAEC dynamic imaging, PAAS coordinate following, Huber sub-pixel fitting, and GBDT correction—is approximately 20 ms. This processing speed seamlessly satisfies the practical 500 ms cycle requirement of modern high-throughput production lines, representing a massive throughput advantage over traditional CMMs.

While the system demonstrates excellent robustness for 820CrMnTi components, its generalizability is subject to certain limitations. The methodology relies heavily on a well-calibrated bi-telecentric optical setup and necessitates initial CMM-measured datasets to adequately train the GBDT error manifold. Furthermore, in scenarios of extreme contamination where heavy oil stains completely occlude the physical edges of the part, the geometric features cannot be recovered physically, potentially compromising the measurement accuracy. Future work will explore cross-material generalization and unsupervised error compensation to further enhance the system’s industrial adaptability.

## 6. Conclusions

To address the challenges of high reflectivity, random poses, and strong noise interference encountered in the industrial online inspection of 820CrMnTi carbonitrided complex-shaped components, this paper proposes a RGFCN that fuses physical-geometric streams with data-driven streams. Through theoretical analysis and experimental verification, the main conclusions of this study are summarized as follows:

(1) Adaptive reconstruction of the physical perception layer achieved high dynamic range imaging. Addressing the non-Lambertian reflection characteristics of the carbonitrided surface, the proposed PAEC mechanism successfully constrained the proportion of high-light pixels to within a 2% threshold through non-linear feedback of the grayscale histogram. This method suppresses information entropy loss caused by highlight overflow at the physical imaging source, laying a signal-to-noise ratio foundation for subsequent high-precision measurements.
(2) The adaptive geometric stream strategy overcame pose limitations in unstructured environments. The Adaptive PAAS strategy eliminates projection cosine errors within a range of ±30° from geometric principles by constructing a following coordinate system. Compared with traditional methods, this strategy increased the Process $C_{pk}$ to 4.73 without requiring precision positioning fixtures, achieving 6-Sigma level process stability and significantly reducing the hardware cost and complexity of production line integration.
(3) The dual-drive heterogeneous architecture achieved statistical immunity to non-functional defects. Targeting common burr and oil stain interference in industrial settings, the Huber M-estimation mechanism automatically identifies and down-weights outliers through a piecewise loss function, effectively avoiding false out-of-tolerance errors caused by the leverage effect in LSE. Combined with the sub-pixel positioning model based on the gradient energy field, the system maintained micron-level measurement precision even under strong noise conditions.
(4) The implicit error compensation mechanism broke through the accuracy bottleneck of traditional geometric optics. GBDT successfully constructed a non-linear mapping relationship for the full-field error, reducing the system residual caused by lens distortion and installation tilt from 0.146 mm to 0.012 mm. Data-driven implicit compensation proves to be an effective approach for resolving non-linear errors in complex optical systems, adding only 3 ms of computational overhead, thus meeting real-time requirements.

## Data Availability

The original contributions presented in this study are included in the article. Further inquiries can be directed to the corresponding author.
